# Sepsis Triggers a Late Expansion of Functionally Impaired Tissue-Vascular Inflammatory Monocytes During Clinical Recovery

**DOI:** 10.3389/fimmu.2020.00675

**Published:** 2020-04-30

**Authors:** Camille Baudesson de Chanville, Benjamin Glenn Chousterman, Pauline Hamon, Marie Laviron, Noelline Guillou, Pierre Louis Loyher, Aida Meghraoui-Kheddar, Sandrine Barthelemy, Philippe Deterre, Alexandre Boissonnas, Christophe Combadière

**Affiliations:** ^1^Sorbonne Université, Inserm, CNRS, Centre d'Immunologie et des Maladies Infectieuses, Cimi-Paris, Paris, France; ^2^Inserm UMRS 1160, Département d'Anesthésie-Réanimation, Hôpitaux Universitaires Lariboisière-Saint-Louis, Paris, France

**Keywords:** monocytes, sepsis, lung, secondary infection, phagocytosis

## Abstract

Sepsis is characterized by a systemic inflammation that can cause an immune dysfunction, for which the underlying mechanisms are unclear. We investigated the impact of cecal ligature and puncture (CLP)-mediated polymicrobial sepsis on monocyte (Mo) mobilization and functions. Our results show that CLP led to two consecutive phases of Mo deployment. The first one occurred within the first 3 days after the induction of the peritonitis, while the second phase was of a larger amplitude and extended up to a month after apparent clinical recovery. The latter was associated with the expansion of Mo in the tissue reservoirs (bone marrow and spleen), their release in the blood and their accumulation in the vasculature of peripheral non-lymphoid tissues. It occurred even after antibiotic treatment but relied on inflammatory-dependent pathways and inversely correlated with increased susceptibility and severity to a secondary infection. The intravascular lung Mo displayed limited activation capacity, impaired phagocytic functions and failed to transfer efficient protection against a secondary infection into monocytopenic CCR2-deficient mice. In conclusion, our work unveiled key dysfunctions of intravascular inflammatory Mo during the recovery phase of sepsis and provided new insights to improve patient protection against secondary infections.

## Introduction

Representing the major cause of admission and death in intensive care units (ICU) (1), sepsis is defined as a life-threatening organ dysfunction caused by a dysregulated host response to an infection (2). Despite adequate treatments, over 20% of septic patients die within 28 days after their admission to the ICU or within the first year after recovery, from secondary infections. Survivors suffer from long-term chronic critical illness often associated with prolonged inflammation, immune suppression, organ injury, and lean tissue wasting (3, 4). It is thought that dysfunctions in both the innate and the adaptive immune system account for the poor outcome in sepsis.

Recent works have focused on understanding how the immune system dysfunctions may contribute to long-term immunosuppression and prolonged sensitivity to secondary infections (4). In the early phase of sepsis, the expansion of immature myeloid cells has attracted much attention (5). They display impaired functions and are reminiscent of myeloid-derived suppressor cells (MDSC) with potent immunosuppressive properties (6). Early sepsis-impaired myeloid functions have been shown to promote nosocomial infections (5, 7). The late phase of sepsis is characterized by T cell exhaustion and a relative increase in regulatory T cells (8, 9), a quantitative and qualitative defect of dendritic cells (10), the deactivation of Mo, demonstrated by the reduced expression of the activation marker HLA-DR (11, 12) as well as the impaired production of cytokines (13). However, not much is known about the kinetic of monocyte (Mo) mobilization throughout sepsis. Two subsets of blood Mo are commonly described in mice and humans: the classical or inflammatory Mo, which are rapidly mobilized upon inflammation in a CC-chemokine receptor 2 (CCR2)-dependent manner, and the non-classical or patrolling Mo that patrols the intraluminal side of the endothelium. In the mouse, inflammatory Mo are short lived, express high levels of Ly6C and CCR2. They are precursors of longer-lived patrolling Mo that lack Ly6C and CCR2 but express higher CX3C-chemokine receptor 1 (CX3CR1) (14). In a previous work, we showed that, soon after the induction of a highly-lethal peritonitis, inflammatory Mo (Ly6C^high^ Mo) migrate from the bone marrow to the blood, adhere in a CX3CR1-dependent way to the endothelium of the renal cortex and protect the kidneys from inflammatory-triggered damages (15). However, the model used in this study does not sufficiently resemble the clinical setting of sepsis in humans, for which the mortality rate is not as elevated. We thus chose to study the distribution, the phenotype and the role of the Mo in a sublethal murine model of peritonitis induced by cecal ligation and puncture (CLP), during the sepsis and following a secondary bacterial infection.

## Materials and Methods

### Mice

All experiments and protocols were approved by the local animal experimentation ethics committee validated by the “Service Protection et Santé Animales, Environnement” with the number APAFIS#4369-2016030218219240 v3. Specific pathogen-free C57BL/6 mice were purchased from Janvier Labs (Le Genest, Saint Isle, France). *Ccr2*^−/−^(#004999, JAX), *Cx3cr1*^−/−^ (16), *Cx3cr1*^*gfp*/*gfp*^ (17), MacBlue or *Csf1r-Gal4VP16/UAS-ECFP* (18), MacBlue x *Cx3cr1*^*gfp*/+^ mice were bred in our animal facility. Age-matched mice (8–12 weeks old) were used for this study.

### Polymicrobial Sepsis Induction

We used a cecal ligation and puncture model as previously described (19). Mice were anesthetized and underwent laparotomy. For Sham-operated mice, the cecum was exteriorized and reinserted in the abdomen. For the CLP-operated mice, sepsis was triggered by the ligature of one third of the cecum and a double enterotomy with a 25-gauge needle. A small amount of fecal material was extruded after removing the needle and the cecum was reinserted in the abdomen. After surgery, the animals were injected with a saline solution and buprenorphine (Vetergesic, Oostkamp, Belgium) for postoperative analgesia. For some experiments, 2 mg/kg of Dexamethasone (Intervet, Beaucouze, France) or 10 mg/kg Enrofloxacine (Axience, Pantin, France) were injected intraperitoneally 24 h after CLP and every 2 for 10 days. Splenectomies were performed prior to CLP procedure. A small upper-quadrant incision was made to expose the spleen. The splenic vessels were tied up and the spleen was removed by transecting the vessels just distal to the ligature.

### *Escherichia coli* Lung Infection

The fluorescent Escherichia coli strain MG1655 ykgH::pTet-dsRed (BGene Genetics, Grenoble, France) was grown overnight in Luria-Bertani (LB) broth (Sigma-aldrich, St Louis, USA) then transferred to fresh medium and grown for 4–5 h to mid-log phase. The OD_600_ was adjusted to give the appropriate desired inoculums, then centrifuged at 4,000 g for 15 min. Bacterial pellets were resuspended in 30 μl of sterile phosphate-buffered saline (PBS) for each sample. To induce secondary *E. coli* lung infection, 10 days after CLP, the trachea was exposed and 30 μl of a bacterial suspension (5 × 10^7^ cfu/mouse for survival studies, 5 × 10^9^ cfu/mouse for adoptive transfer experiments, or 1 × 10^7^ cfu/mouse for all other studies) or sterile PBS were administered intratracheally to sham- or CLP-operated mice 24 and 48 h before sacrifice. This procedure was performed under Ketamine/Xylazine anesthesia.

### Adoptive Transfer Experiments

Bone marrow cells were isolated 10 days after CLP or sham procedure in WT mice. Mo were isolated after negative selection removal of other cell types, with Ly6G, CD3, CD4, CD19, NK1.1, and SiglecF-PE labeled antibodies. Marked cells were then captured via a magnetic device for cell separation and anti-PE magnetic beads, according to the manufacturer's instructions (Miltenyi Biotec, Paris, France). Thirty million monocytes were injected intravenously in *Ccr2*^−/−^ mice, and *E.coli* (5 × 10^9^ cfu/mouse) were injected intratracheally 30 min later. The proportions of Mo adoptively transferred from each condition were controlled before transfer by flow cytometry and were identical. Mo represented between 12 and 16% of myeloid cells and were enriched by 70–80% after sorting. PMN population was <1%. Mice were monitored every 12 h for survival and surviving mice were used for quantification of protein in lung homogenates at day 4.

### Bronchoalveolar Lavage (BAL) and Bacterial Load

BAL were performed on mice 48 h after the secondary lung injection. The BAL performed with 3 ml of sterile PBS was diluted and plated on LB agar plates to obtain viable bacterial counts (cfu/BAL).

### Cell Isolation and Preparation

Heparinized blood samples were stained with antibodies and erythrocytes were lysed with buffer containing 0.15M NH4Cl, 0.01 mM KHCO3, and 0.1 mM EDTA. Bone marrow cells were harvested by flushing out the thighbone with PBS. Lung, spleen, kidney, and liver were harvested and digested in RPMI medium (Gibco, Invitrogen, Cergy Pontoise, France) with 1 mg/ml collagenase IV (Sigma, St Quentin Fallavier, France), 0,1 mg/ml DNAse 1 (Roche, Boulogne Billancourt, France) for 30 min at 37°C and dissociated through a 40-μm-pore cell strainer (Becton Dickinson, Rungis, France). Diluted suspension cells were incubated with 1 μg/ml purified anti-CD16/32 (clone 2.4G2, BD Biosciences) for 10 min at 4°C then surface staining was performed with an additional 20 min incubation with appropriate dilution of the surface marker antibodies. Cell suspensions were washed once in FACS buffer (0.5% BSA, 2 mM EDTA and PBS) and analyzed directly by flow cytometry.

### Blood/Tissue Partitioning

Intravascular CD45 labeling was performed as previously described (20, 21). Mice were injected intravenously with 2 μg of anti-CD45 (clone 30-F11, BD Biosciences). Two minutes after injection, blood was drawn and the mice were sacrificed. Harvested organs were bathed in a large volume of PBS. CD45 labeled cells in all tissues were considered to be intravascular (CD45vivo+) and unlabelled cells (CD45vivo-) were considered to be parenchymal.

### Flow Cytometry

The panel of antibodies comprised: BUV395-CD11b (clone M1/70), APC-Cy7-Ly6C (clone AL21), V450-Ly6G (clone 1A8), BV711-NK1.1 (clone PK136), BV605-CD11c (clone HL3), BV510-I-A/I-E (clone M5/114.15.2), BV786-SiglecF (clone E50-2440), BUV737-CD80 (clone 16-10A1), APC-R700-CD86 (clone GL1), APC-PDL1 (clone MIH5), APC-IL6 (clone MP5-20F3), BV421-TNFα (clone MP6-XT22), which were from BD Biosciences, and FITC-CX3CR1 (clone SA011F11) and PE-Cy7-CD64 (clone X54-5/7.1), which were from Biolegend. Relative changes in cytosolic nitric oxide (NO) concentration were monitored using the fluorescent nitric oxide probe DAF-FM (Molecular Probes, Eugene, OR). The cells extracted from the lungs were incubated with DAF-FM diacetate (5 μM) for 30 min at 37°C. After an extensive wash, cells were stained with fluorescent surface antibodies and NO production was measured in monocytes by flow cytometry. For IL-6 and TNFα staining, cells were preincubated for 3 h at 37°C in RPMI medium supplemented with GlutaMAX with a cell activation cocktail containing Brefeldin A according to the manufacturer's instructions (BioLegend). After surface staining, the cells were fixed in 4% paraformaldehyde for 20 min, washed twice in Perm/Wash solution (BD Biosciences), incubated for 10 min with 1 μg/mL purified anti-CD16/32 in Perm/Wash at room temperature, and incubated for 30 min in Perm/Wash in the presence of APC-anti-IL-6 (BD Pharmingen) or BV421-anti-TNFα (BD Horizon). Flow cytometry acquisition was performed on the flow cytometer FACS LSRFortessa X-20^®^ (BD, Franklin Lakes, NJ, USA) with DIVA^®^ Flow Cytometry software, and the data was analyzed with FlowJo software (Tree Star, Inc, Ashland, Or, USA). Absolute numbers were calculated by adding to each vial a fixed number (10,000) of non-fluorescent 10-μm polybead^®^ carbocylate microspheres (Polysciences, Niles, IL, USA) according to the formula: No. Cells = (No. acquired cells x 10,000)/(No. acquired beads) x dilution factor of cells.

### *In vitro* Cell Stimulation

Lung cell suspensions (3 × 10^5^ cells from Sham mice or 3 × 10^4^ cells from CLP-operated mice) were plated in 96 well plates and stimulated with either Cell Activation Cocktail PMA/Ionomycin at 1X (Biolegend, San Diego, USA) or 2 ng/ml of LPS (Sigma-Aldrich, St Louis, USA) in RPMI containing 10% FBS for 3 h at 37°C with 5% CO_2_. Cells were recovered and washed with fresh PBS 1X and then stained for flow cytometry analysis.

### Phagocytosis Assay

For *in vivo* phagocytosis, a total of 1 × 10^7^ cfu/mouse of DS-Red fluorescent *Escherichia coli* were intratracheally injected 10 days after CLP surgery (as described previously). Phagocytosis by lung cells was analyzed 24 and 48 h after infection by flow cytometry. The fluorescence of phagocytic cells was also observed in histological sections. Control mice were injected with sterile PBS under the same experimental conditions. *In vitro* phagocytosis was performed with lung cells suspension obtained 10 days after CLP mixed with 5 × 10^5^ cfu of DS-Red fluorescent *Escherichia coli* at a 1:5 ratio (cells/bacteria) during 4 h at 37° or 4°C for the control experiment. Percentage of Ly6C^high^ Mo phagocytosis was determined by flow cytometry. In the 4°C control condition, phagocytosis index was <3%.

### RNA Extraction and Quantitative Real-Time PCR

Lungs were harvested 48 h after the E. coli lung infection 10 days post CLP. Cells were isolated as described above. Total RNA was extracted using the RNeasy Mini Kit (QIAGEN, Les ulis, France) according to the manufacturer's instructions. RNA concentration was determined by absorption at 260 nm. cDNA synthesis was performed with SuperScript VILO cDNA Synthesis Kit (Invitrogen). The polymerase chain reaction was performed on an ABI prizm 7300 using Power SYBR Green PCR Master Mix (Life technologies, California, USA) and GAPDH was used as the control gene. Primers for iNOS: F-CCAAGCCCTCACCTACTTCC; R-CTCTGAGGGCTGACACAAGG, IL-4: F- CCATATCCACGGATGCGACA; R- AAGCCCGAAAGAGTCTCTGC, IL-10: F- GCTCTTACTGACTGGCATGAG; R- CGCAGCTCTAGGAGCATGTG, IL-6: F- CGGCCTTCCTACTTCACAA; R- GGTACTCCAGAAGACCAGAGGA, TGFB: F- atgctaaagaggtcacccgc; R- GTATCAGTGGGGGTCAGCAG, CCL2: F- CCCCACTCACCTGCTGGTA; R- TTACGGGTCAACTTCACATTCAAA.

For transcript-level analysis, results were expressed as a fold increase relative to sham condition at day 10.

### Multi-Photon Imaging

Freshly explanted lungs were immobilized in an imaging chamber perfused with oxygenated (95% O2 plus 5% CO_2_) RPMI medium containing 10% FCS. The local temperature was monitored and maintained at 37°C. For some experiments, 10 μg of anti-CD31 (AF647; clone 390) were injected intravenously 2 min before euthanasia. The Two-Photon Laser Scanning Microscopy (TPLSM) set-up used was a Zeiss 7MP (Carl Zeiss, Germany) coupled to a Ti:Sapphire Crystal multiphoton laser (Coherent ChameleonU, CA, USA) which provides 140fs pulses of NIR light, selectively tunable between 680 and 1,050 nm and an optical parametric oscillator (OPO-MPX, Coherent) selectively tunable between 1,050 and 1,600 nm. The system included a set of external non-descanned detectors in reflection with a combination of a LP-600 nm followed by LP-462 nm and LP-500 nm dichroic mirrors to split the light and collect the ECFP with a 480/40 nm emission filter, EGFP with a 525/50 nm emission filter. The excitation wavelength was 870 nm for the NLO beam and 1,100 nm for the OPO beam.

### Lung Protein Quantification

The mouse lung vasculature was gently flushed with an intracardiac injection of PBS until complete blood clearance, then lungs were collected and crushed in 1 ml of PBS. The supernatant of pulmonary crushed tissue was used to quantify protein level by enzymatic assay, BCA protein assay (Pierce, Waltham, USA) according to the manufacturer's standard protocol.

### Data Presentation and Statistical Analysis

The data are presented as mean ± standard error of the mean (s.e.m.) of the indicated number of experiments. Groups were compared with Prism software (Graphpad, San Diego, USA). Statistical analyses were performed using two-tailed Student's *t*-test for two-group comparisons, or one-way and two-way ANOVA tests with Bonferoni multiple comparison tests: ^*^ for *p* < 0.05; ^**^ for *p* < 0.01; ^***^ for *p* < 0.001; ^****^ for *p* < 0.0001. Kaplan-Meier survival curves were compared with a log-rank test, where *p* < 0.05 was considered statistically.

## Results

### Polymicrobial Sepsis Induces Two Distinct Phases of Monocyte and of Neutrophil Deployment in the Blood and Organs

**Figure 1:**
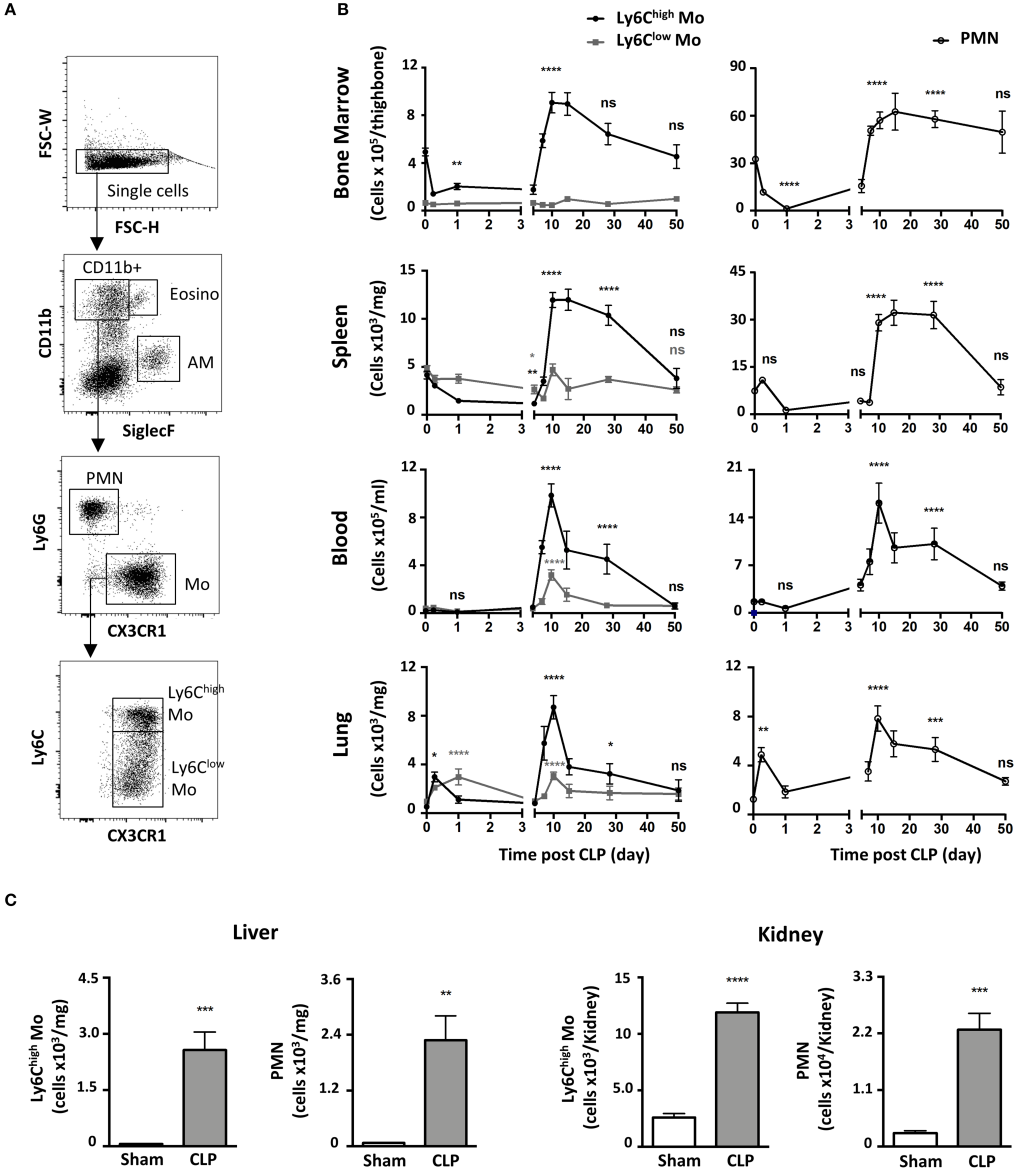
Polymicrobial sepsis induces two distinct phases of Mo and of neutrophil deployment in the blood and organs. **(A)** Representative flow cytometry gating strategy for myeloid subsets in the lung: Eosinophils (Eosino), Alveolar Macrophages (AM), Polymorphonuclear Neutrophils (PMN), classical Mo (Ly6C^high^ Mo), non-classical Mo (Ly6C^low^ Mo). **(B)** Ly6C^high^, Ly6C^low^ Mo, and PMN numbers in the bone marrow, spleen, blood, and lungs as determined by flow cytometry at different time points after CLP. Tissue-resident macrophages were excluded from analysis based on CD64 expression. The baseline was defined on the cell number obtained in sham-operated mice. Each time point represents at least three independent experiments run with 6 to 12 mice. Statistical analyses compare the different time points after CLP to baseline. **(C)** Numbers of Ly6C^high^ Mo and PMN in the liver (left panel) and the kidneys (right panel), 10 days after sham- (white) and CLP-operated (gray) mice. Values represent the mean and standard error of the mean (sem) of 6 mice per group from two independent experiments. Unpaired Student's *t* test was performed. ^*^p < 0.05; ^**^p < 0.01; ^***^p < 0.001; ^****^p < 0.0001.

We characterized the myeloid composition of the blood and organs in a mouse model of peritonitis induced by cecal ligation and puncture (CLP) which results in polymicrobial sepsis and inflammation (15, 19). In the chosen conditions, 100% of the mice suffered from severe weight loss (Supplementary Figure 1A) and about 10% of the mice succumbed in the first 4 days (Supplementary Figure 1B). For surviving mice, normal weight was almost recovered within 10 days following surgery (Supplementary Figure 1A). Mo subsets and PMN distributions were analyzed by flow cytometry using a conventional gating strategy (Figure 1A) to identify classical Mo named here Ly6C^high^ Mo (defined as CD11b^+^/Ly6G^−^/CX3CR1^+^/Ly6C^high^), non-classical Mo named here Ly6C^low^ Mo (defined as CD11b^+^/Ly6G^−^/CX3CR1^+^/Ly6C^low^) and PMN (defined as CD11b^+^/Ly6G^+^/CX3CR1^−^). Eosinophils were excluded by SiglecF expression, Natural Killer cells by NK1.1 and alveolar macrophages by siglecF and CD64 (not shown). During the acute phase, a few hours after sepsis induction (Figure 1B, left panels), Ly6C^high^ Mo numbers decreased in the bone marrow (upper panels) and the spleen (upper middle panel) and remained low until day 5. During this period, Ly6C^low^ Mo numbers remained fairly constant in these tissues. The numbers of blood Ly6C^high^ and Ly6C^low^ Mo remained stable for the first few days after sepsis (lower middle panel) whereas both Mo subpopulations accumulated rapidly in the lungs (day 1–2; first wave) as previously observed in the kidney (15), and returned to baseline by day 5 (lower panel). Along with mice weight recovery (a week after CLP induction), the numbers of Ly6C^high^ Mo increased dramatically in all tissues; bone marrow (~2-fold), blood (~50-fold), spleen (~3-fold) and lungs (~20-fold) peaking between day 10 and 15 and returning back to sham values only by day 50. Again, variations in the number of Ly6C^low^ Mo were modest during this time period (7–50 days) compared to that of Ly6C^high^ Mo. PMN underwent a kinetic of mobilization similar to that of Ly6C^high^ Mo in all four studied tissues (Figure 1B, right panels). Between 4 and 10 days, the number of alveolar macrophages dropped massively and recovered thereafter (Supplementary Figure 1C). Strong accumulations of both Ly6C^high^ Mo and PMN were also observed during weight recovery in the kidneys (~5 and 10-fold, respectively) and in the liver (~40 and 30-fold, respectively) arguing for a systemic accumulation of Mo and PMN in non-lymphoid tissues (Figure 1C). Globally, polymicrobial sepsis induced two distinct phases. The “early acute phase” is characterized by extensive weight loss and death. This phase is associated with a massive Mo and PMN mobilization to the lungs correlating with a deep draining of the myeloid tissue reservoirs, the bone marrow and the spleen. The second phase is characterized by clinical improvements and weight recovery. This “recovery phase” is associated with Mo and PMN expansion in the tissue reservoirs, release in the blood and accumulation in peripheral non-lymphoid tissues including the lungs, liver and kidneys.

### Ly6C^high^ Mo Deployment During the Recovery Phase Requires Inflammatory Pathways

**Figure 2:**
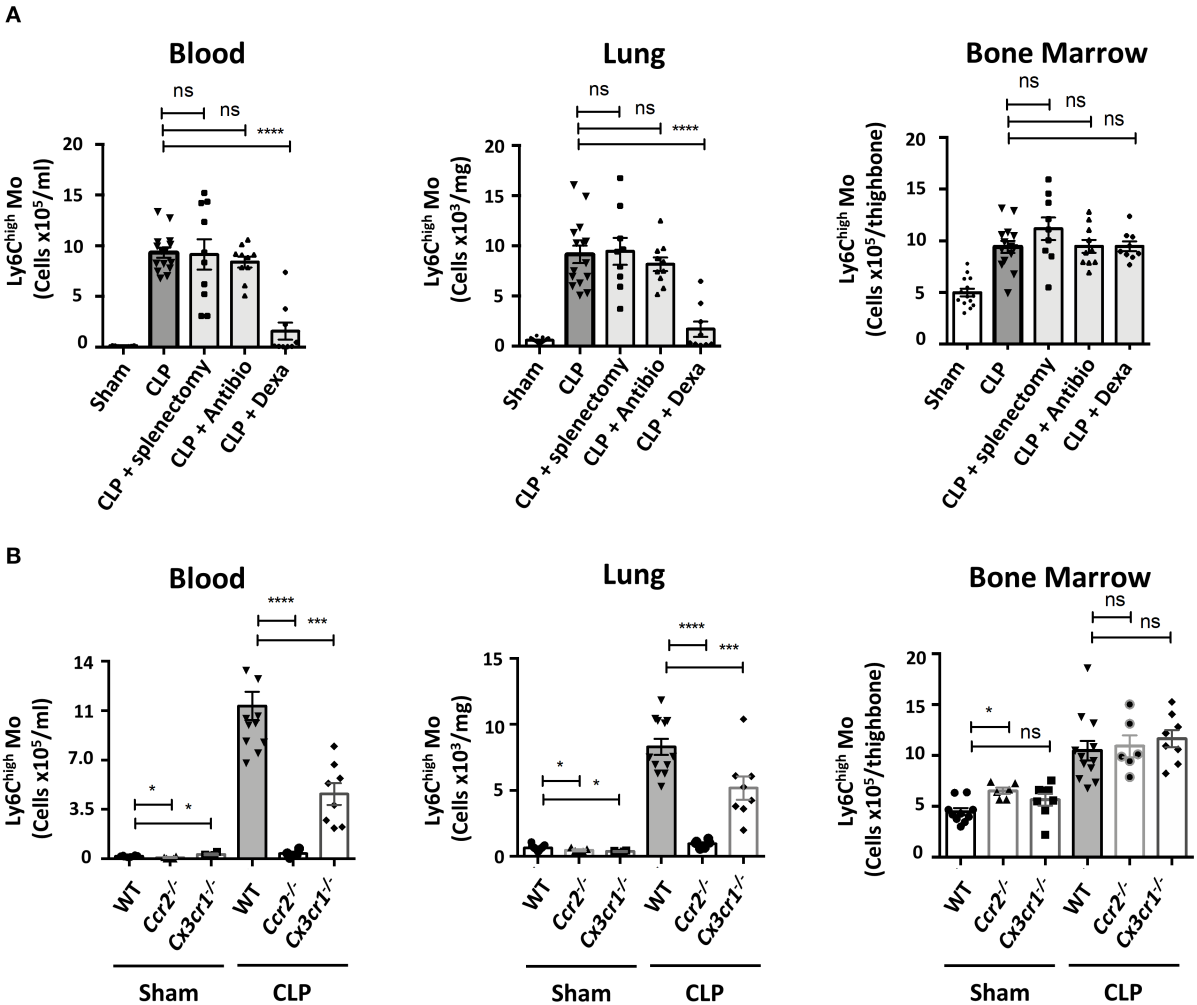
Ly6C^high^ Mo deployment during the recovery phase requires inflammatory pathways. **(A)** Numbers of Ly6C^high^ Mo in the blood, lungs and bone marrow determined by flow cytometry in sham and septic mice, 10 days after CLP and treatments. Splenectomies were performed a few minutes before the CLP. Antibiotic or Dexamethasone treatments were injected 24 h after CLP. Values represent the mean and standard error of the mean of 9–14 mice per group from two independent experiments. Two-way ANOVA tests with Bonferoni multiple comparison tests compare each group (light gray) to the CLP controls (dark gray); ^****^ for *p* < 0.0001. **(B)** Numbers of Ly6C^high^ Mo in the blood, lungs, and bone marrow determined by flow cytometry, 10 days after sham- and CLP-operated WT (*n* = 12), *Ccr2*^−/−^ (*n* = 6), and *Cx3cr1*^−/−^ (*n* = 8) mice from at least two independent experiments. Two-way ANOVA tests with Bonferoni multiple comparison tests compare chemokine receptor deficient mice to the WT mice, ^*^ for *p* < 0.05; ^***^ for *p* < 0.001; ^****^ for *p* < 0.0001.

We looked into characterizing what drives Mo and PMN redistributions during weight recovery. Expanded populations of both Mo and PMN in septic conditions are thought to originate from bone marrow precursors (22), but in inflammatory conditions the spleen can develop extra medullary myelopoiesis (23). The accumulation of Ly6C^high^ Mo in blood, lungs and bone marrow were similar in mice both splenectomized or not (Figure 2A). These data indicate that the spleen is dispensable in CLP-elicited Mo deployment and suggest that this phenomenon may rely solely on bone marrow. Because both infections and inflammation are known to elicit changes in myelopoiesis and mobilization, CLP-operated mice were treated with antibiotics or anti-inflammatory drugs. The broad-spectrum antibiotic treatment did not alter the late accumulation of either Ly6C^high^ Mo nor PMN in the different tissues (Figure 2A and Supplementary Figure 2A). Conversely, anti-inflammatory treatment with Dexamethasone abrogated the CLP-triggered accumulation of Ly6C^high^ Mo, Ly6C^low^ Mo (Supplementary Figure 2B) and PMN (Supplementary Figure 2A) in the blood and the lungs but had no effect on bone marrow. We next thought to identify inflammatory chemokine receptors leading to Mo mobilization (Figure 2B). As previously observed, *Ccr2*^−/−^ or *Cx3cr1*^−/−^ sham operated mice displayed less classical Mo in the lungs (20, 24). In septic mice, monocytosis was totally or partially reduced in the blood and the lungs of *Ccr2*^−/−^ or *Cx3cr1*^−/−^ mice, respectively. The absolute number of the Ly6C^high^ Mo in the bone marrow remained unaffected by these chemokine receptor deficiencies (Figure 2B). CCR2 and CX3CR1 deficiency did not affect PMN or alveolar macrophage numbers compared to WT mice (Supplementary Figures 2C,D), confirming the selective role of these two chemokine axes in the regulation of Mo deployment to the lungs during the recovery phase.

### Late-Expanded Mo Display an Altered Phenotype and Remain Intravascular in the Lungs of Septic Mice

**Figure 3:**
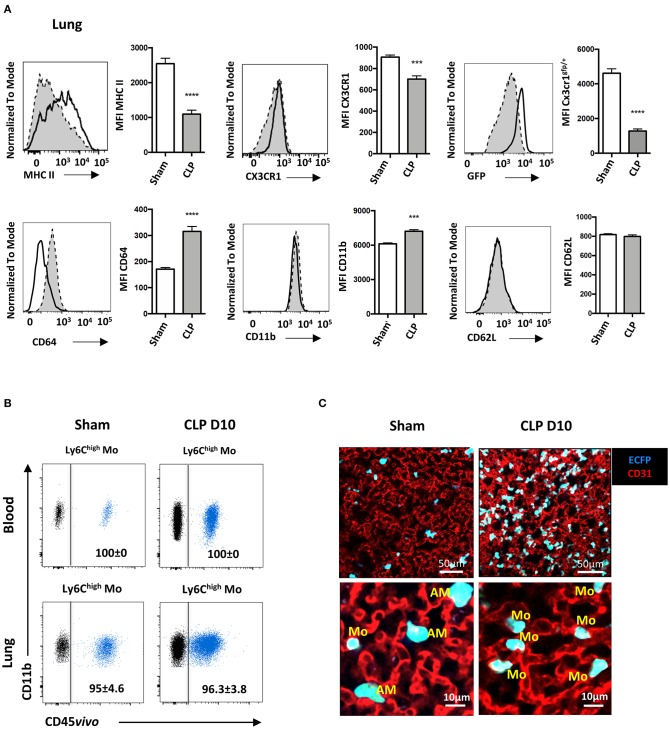
Late-expanded Mo display an altered phenotype and remain intravascular in the lungs of septic mice. **(A)** Overlay of flow cytometric surface marker expression gated on Ly6C^high^ Mo in the lungs of sham- (black line) and CLP-operated (dotted line) mice, 10 days after CLP. Histograms represent mean fluorescence intensity (MFI) of surface marker expression: MHC-II, CX3CR1, GFP, CD64, CD11b, and CD62L. Values represent the mean and standard error of the mean of 15 sham- and 12 CLP-operated mice from three repeated experiments. The GFP fluorescent reporter of Cx3cr1 expression was performed on 9 sham- and 9 CLP-operated *Cx3cr1*^*gfp*/+^ mice. Unpaired Student's *t* test was performed. **(B)** Representative overlayed dot plots of *in vivo* CD45 staining (blue) gated on blood (upper panels) and lung (lower panels) of Ly6C^high^ Mo. Background staining shown in black was measured in mice not injected with the anti-CD45. **(C)** Representative images of two-photon microscopy of explanted lungs from sham- or CLP-operated MacBlue x Cx3cr1^gfp/+^ mice. Lung vasculature is visualized using anti-CD31 staining (red), and Mo were observed using ECFP reporter. Inserts represent a 5x zoom showing a few alveolar macrophages (AM) in the alveolar lumen (black space) in a sham-operated mouse and intravascular Mo in a CLP-operated mouse. ^***^p < 0.001; ^****^p < 0.0001.

We next characterized the phenotype of the Mo accumulating into the lungs during the recovery phase of sepsis. Ten days after CLP (Figure 3A), MHC class II expression on Ly6C^high^ Mo was reduced by more than 50%, as previously reported (11, 12). CX3CR1 expression was reduced by 20% compared to sham-operated mice, whereas expression of the EGFP reporter from *Cx3cr1*^*egfp*/+^ mice was severely reduced, as previously reported (25, 26). Conversely, CD64 expression doubled on Ly6C^high^ Mo from CLP-operated mice and CD11b expression was modestly increased. CD62L expression remained unchanged. In order to further investigate the tissue localization of Mo in the lung, we performed blood/tissue partitioning using *in vivo* CD45 labeling (20). All blood Mo (Figure 3B, upper panels) and more than 95% of lung Mo (Figure 3B, lower panels) were strongly positive for CD45 staining either in sham- or CLP-operated mice, indicating that the Mo reside exclusively in the lung vasculature and did not infiltrate the tissue even in septic mice. Similar results were obtained with Ly6C^low^ Mo (Supplementary Figure 3A) and PMN (Supplementary Figure 3B). In the MacBlue transgenic mouse, the *Csf1r* promoter lacks the 150 bp trophoblast and osteoclast-specific transcription start sites, driving the expression of ECFP on classical and non-classical Mo, alveolar macrophages with a lower intensity and a fraction of granulocytes, but not in lung interstitial macrophages (18, 20). Imaging fresh explanted lungs of sham- and CLP-operated MacBlue mice using multiphoton microscope after *in vivo* labeling of the vasculature using fluorescent anti-CD31 (Figure 3C), confirmed their anatomical localization. In the lungs of sham-operated MacBlue mice, a few small cyan round-shaped Mo (ECFP^+^) were detected in the vasculature (upper left picture and 5X magnification below) escorted by larger round-shaped ECFP+ alveolar macrophages that stayed exclusively in the alveolar lumen. In septic mice, ECFP+ Mo strongly accumulated within the lung capillaries (upper right picture and magnification below) whereas alveolar macrophages were barely detectable (Figure 3C), in accordance with the flow cytometry data (Supplementary Figure 1). Both flow cytometry and microscopy analyses revealed that during the recovery phase of sepsis, inflammatory Mo with altered expression of cell activation markers, such as MHC class II, CX3CR1 and CD64, invaded the lungs (and probably the kidneys and liver) but remained fully intravascular. They do not infiltrate the parenchymal and stromal tissues, nor the alveolar space.

### Mo of the Late Recovery Phase of Sepsis Display an Altered Phenotype and Fail to Protect From Secondary Infection

**Figure 4:**
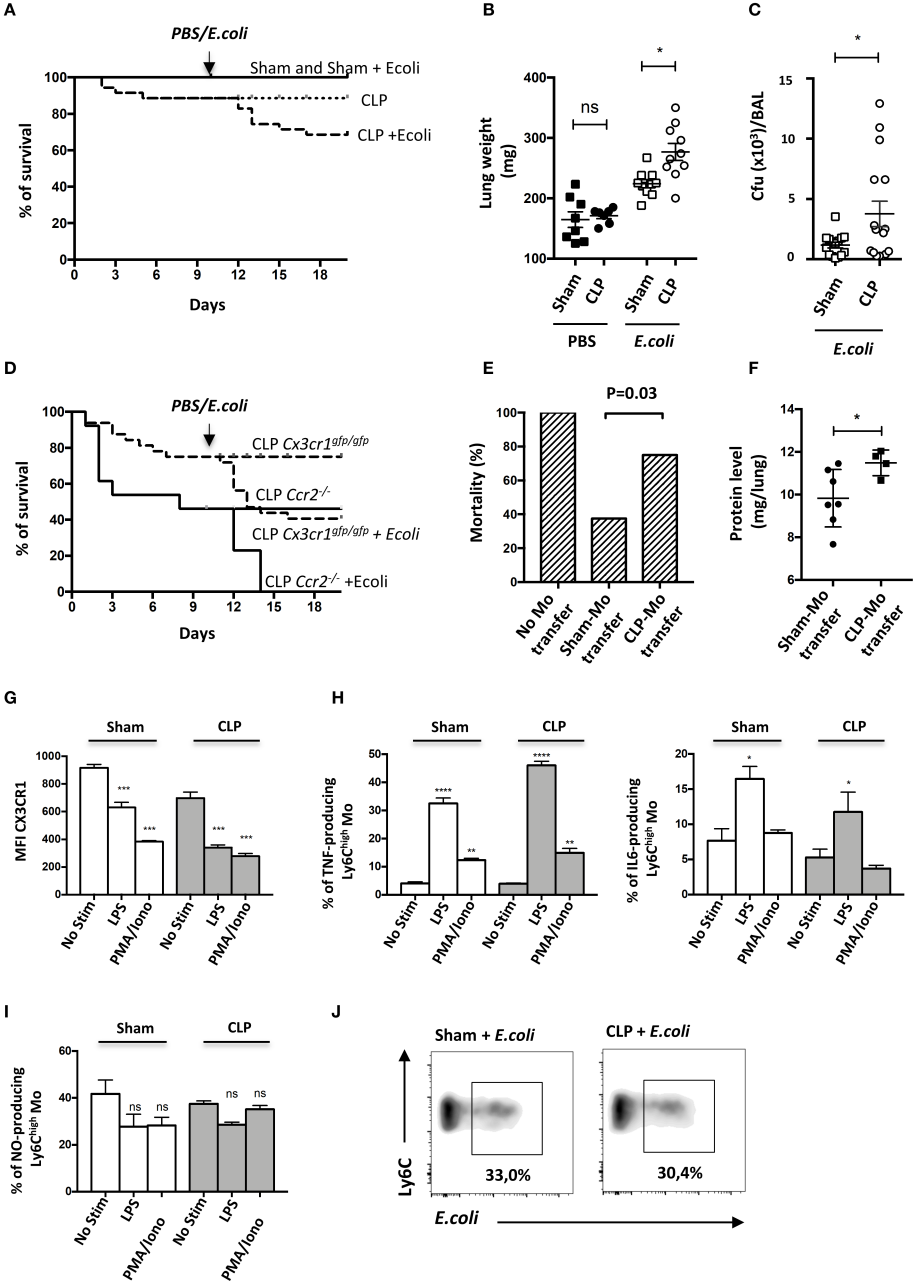
Mo of the late recovery phase of sepsis display an altered phenotype and fail to protect from a secondary infection. **(A)** Survival of sham- or CLP-operated *Cx3cr1*^*gfp*/+^ mice with intratracheal injection of *Escherichia coli* on day 10 after surgery (*n* = 8–12 per group). **(B)** Total lung weight of sham- and CLP-operated C57Bl6 mice at 48 h after *E. coli* secondary infection. Unpaired Student's *t* test was performed. **(C)** Bacterial colonization of bronchoalveolar lavages (BAL) from sham- and CLP-operated C57Bl6 mice at 48 h after *E. coli* secondary infection. Unpaired Student's *t* test was performed. **(D)** Survival of sham^−^ or CLP-operated *Cx3cr1*^*gfp*/*gfp*^ (*n* = 12 per group) and *Ccr2*^−/−^ (*n* = 8–10 per group) mice with intratracheal injection of *Escherichia coli* on day 10 after surgery. **(E)** Histogram represents the percentage of death in *Ccr2*^−/−^ mice after transfer of purified Mo from Sham- or CLP-operated mice at 4 days after *E. coli* infection 5 × 10^9^ cfu/mouse (*n* = 16 mice from three repeated experiments). *p* was determined using a chi-square test between the mice that received adoptive transfer. **(F)** Protein quantification in lung homogenates after adoptive transfer and E.coli pulmonary infection in *Ccr2*^−/−^ surviving mice. Unpaired Student's *t* test was performed. **(G)** Mean fluorescence intensity (MFI) of CX3CR1 surface marker of lung Ly6C^high^ Mo from sham- and CLP-operated mice at day 10. Cells are stimulated or not with LPS or PMA-Ionomycin *in vitro*. Values represent the mean +/– Sem of 10 mice from two repeated experiments. **(H)** Percent of TNFα-(left panel) or IL-6-(right panel) producing lung Ly6C^high^ Mo (left panel) from sham- and CLP-operated mice at day 10. Values represent the mean +/– Sem of 4–6 mice. One-way ANOVA tests with Bonferoni multiple comparison tests compare Sham and CLP, respectively. **(I)** Percent of NO-producing lung Ly6C^high^ Mo from sham- and CLP-operated mice at day 10. Values represent the mean +/– Sem of 4–6 mice. One-way ANOVA tests with Bonferoni multiple comparison tests compare Sham and CLP, respectively. **(J)** Representative dot plot (out of 8 mice from two repeated experiments) of phagocytic Ly6C^high^ Mo percentage, 4 h after *in vitro* co-culture between lung cells suspension and fluorescent *E. coli*. ^*^p < 0.05; ^**^p < 0.01; ^***^p < 0.001; ^****^p < 0.0001.

Septic patients have a higher risk of developing secondary nosocomial infections (27). To determine whether mice are more susceptible to pneumonia after sublethal CLP, they were infected with E. coli by intratracheal injection (10 days post-CLP) and the survival rates of each group were evaluated (Figure 4A). Sham-operated mice were fully resistant to the second infection whereas about 20% of septic mice died during the first week following the bacteria inoculation. This excess of mortality was associated with an increased lung weight (Figure 4B) and bacterial lung load in bronchoalveolar lavage (BAL) (Figure 4C). These results confirm that sepsis triggers an increased susceptibility and severity to secondary infections. Because Mo recruitment is associated with disease severity in chronic inflammation (28), we used *Ccr2*^−/−^ and *Cx3cr1*^*egfp*/*egfp*^ to study the impact of Mo mobilization in response to the secondary infection. *Ccr2*^−/−^ mice were more likely to die after CLP with about 46% mortality by day 10 and succumbed rapidly to secondary infection with 100% of death by day 4 post-secondary infection (Figure 4D). *Cx3cr1*^*egfp*/*egfp*^ mice also displayed increased mortality in both primary (20%) and secondary infection (40%). Susceptibility to secondary infection was thus inversely proportional to the extent of Mo expansion observed in CCR2- and CX3CR1-deficient mice, suggesting a protective role of Mo in these infectious conditions. We postulated that Mo accumulated in the vasculature of septic mice may not be as efficient to protect from secondary infection as Mo from control mice. Adoptive transfer in *Ccr2*^−/−^ mice is a model to evaluate the functional role of Mo (29, 30). We thus compared the survival of *Ccr2*^−/−^ mice to lethal E.coli infection after Sham- and CLP- Mo adoptive transfer. In *Ccr2*^−/−^, E.coli infection led to 100% of mortality after 4 days (Figure 4E). Adoptive transfer of Mo purified from sham-operated mice rescued about 62% of the mice, whereas only 25% were rescued after being transferred with Mo purified from CLP-operated mice. Coincidently, protein levels in lung homogenates of surviving mice were higher in mice transferred with Mo purified from CLP-operated mice compared to those of mice that received Mo purified from Sham-operated mice (Figure 4F), possibly indicating an increased vessel permeability due to increased microvascular lung lesions. In order to assess endotoxin tolerance (31) that may render cells unresponsive when re-challenged with lipopolysaccharide (LPS) produced by fecal microbiota induced by the CLP surgery, Mo purified from either sham- or CLP-operated mice were challenged *ex vivo* with LPS (Figures 4G–I). As predicted, it triggered a strong downmodulation of the CX3CR1 (Figure 4G) on Mo from sham-operated mice, as shown in Figure 3A. The chemical stimulation (PMA/ionomycine) used as an activation that is TLR-independent also triggered a strong downmodulation of CX3CR1. LPS triggered a strong production of TNFa and IL-6 (Figure 4H) in Mo from both sham- and CLP-operated mice in comparison to PMA/ionomycine stimulation. Nitric oxide (NO) production by Ly6C^high^ Mo was measured using a photo-stable fluorescent probe named DAF-FM. No additional NO production was induced after LPS or PMA/ionomycine stimulation. Globally, LPS stimulation on Mo from CLP-operated mice was similar to that observed on Mo from sham-operated mice, indicating that Mo from CLP-operated mice were still responsive to LPS. In addition, Mo were challenged *ex vivo* with E. coli fluorescently labeled for phagocytic activity that was measured in a flow cytometry assay (Figure 4J). Again, lung Mo from CLP-operated mice (and thus exposed to fecal *E. coli*) were as efficient as Mo from sham-operated mice to phagocyte bacteria *in vitro*.

### Primary Sepsis Hampers Mo Activation Without Altering Mo Infiltration

**Figure 5:**
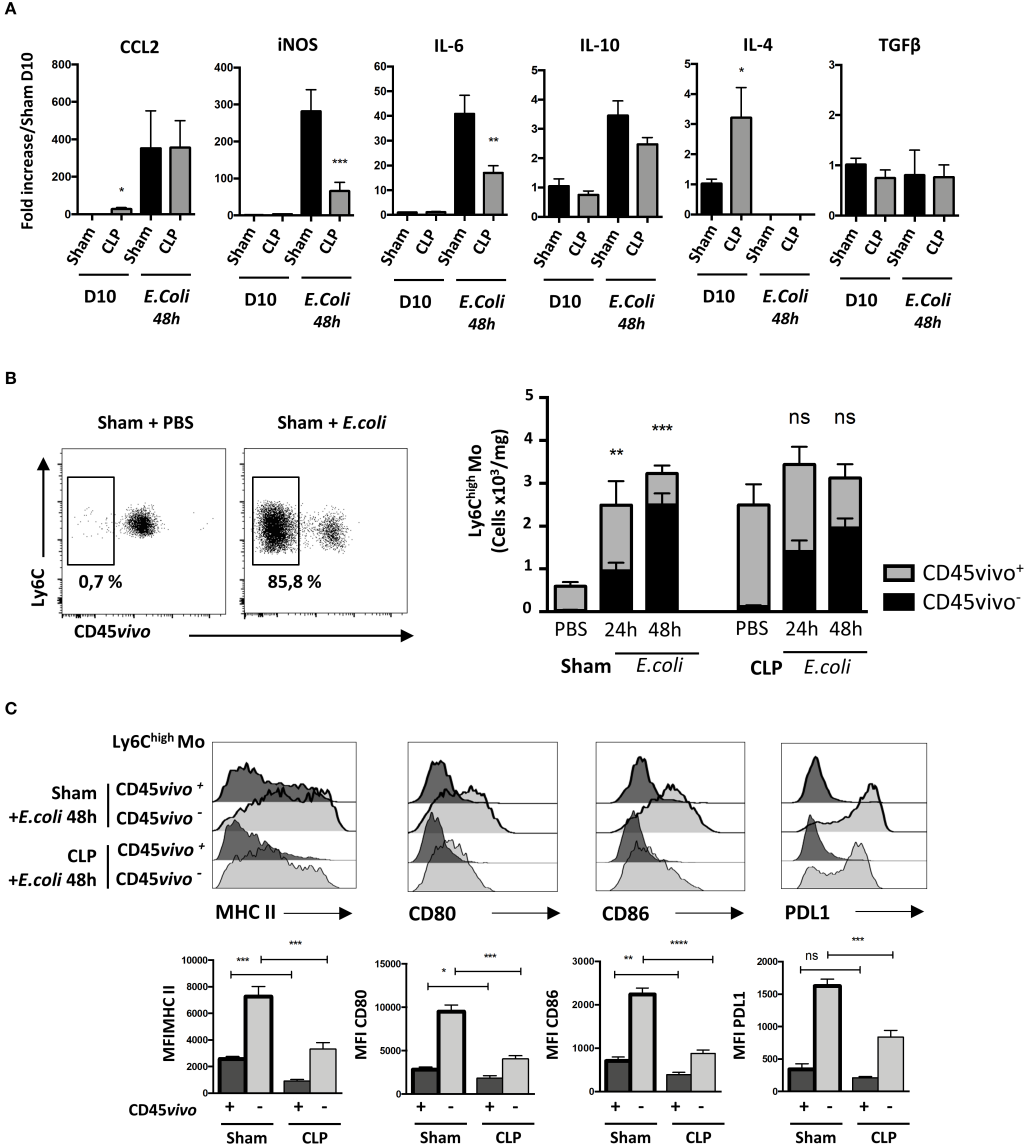
Primary sepsis hampers Mo activation without altering Mo infiltration. **(A)** mRNA levels in lung cells measured by qPCR compared to sham-operated mice. Unpaired Student's *t* test was performed to compare Sham and CLP-operated mice in presence or absence of *E. coli*. **(B)** The left panels are representative dot plots of lung infiltrated Ly6C^high^ Mo from sham-operated mice injected with PBS or after *E. coli* infection. The histogram (right panels) represents the total numbers of Ly6C^high^ Mo per mg of lung from sham- or CLP-operated mice, 24 and 48 h after *E. coli* infection or PBS injection. Black bars represent the CD45vivo^−^ infiltrated cells and gray bars are CD45vivo^+^ vascular cells. Values represent the mean +/– sem of 10 mice per group. **(C)** Overlay of flow cytometric surface marker expression gated on lung vascular (CD45vivo ^+^) and infiltrated (CD45vivo ^−^) Ly6C^high^ monocytes in sham- (thick black lines) and CLP- (thin black line) operated mice, 48 h post E. coli infection. Mean fluorescence intensity (MFI) of CMH II, CD80, CD86, and PDL1 surface markers on infiltrated CD45vivo^−^ (light gray) or vascular CD45vivo^+^ (dark gray) Ly6C^high^ Mo in the lungs of sham- (thick black lines) and CLP- (thin black line) operated mice 48 h after second infection. ^*^p < 0.05; ^**^p < 0.01; ^***^p < 0.001; ^****^p < 0.0001.

We then analyzed transcript levels of chemokines and cytokines in the whole lung to identify potential alterations leading to increased susceptibility and severity to secondary infections in septic mice (Figure 5A). Ten days after surgery, only *Ccl2* and *Il4* transcript levels were statistically more abundant in the lungs of CLP-operated mice compared to those of sham-operated mice. *E. coli* infection triggered a massive increase in transcript levels for Ccl2, i*Nos* or *Il6*, a modest increase in *Il10* transcripts but totally abrogated *IL4* transcript expression in both Sham- and CLP-operated mice. It had no effect on *Tgfß* transcripts. *Ccl2* transcript levels in septic mice were unaffected by *E. coli* injection. Interestingly, the lungs of septic mice had less abundant transcripts for *iNos* and *Il6* than sham-operated mice. We thought that the altered cytokine environment observed 10 days after CLP may be associated with a defect in Mo deployment. We studied the distribution of Ly6C^high^ Mo between the lung vasculature and the parenchyma after *E. coli* infection using CD45 intravascular staining (Figure 5B, left panel). In sham-operated mice, *E. coli* injection triggered a strong accumulation of vascular (CD45vivo^+^) Ly6C^high^ Mo and a strong tissue infiltration (CD45vivo^−^) at 24 h post E. coli infection (Figure 5B, right panel). Although Mo did not accumulate further between 24 and 48 h, the relative proportion of Mo that infiltrated the lung increased. As previously described in Figure 1, septic mice at day 10 post-CLP already displayed increased numbers of Mo residing in the lung vasculature compared to Sham-operated mice before the second infection. Forty-eight hours after E. coli infection, no additional Mo accumulation was detected when compared to CLP-operated mice injected with PBS. Mo from septic mice extravasated in the lung parenchyma (CD45vivo^−^) in similar proportion than Mo from sham-operated mice, suggesting that sepsis does not alter the capacity of Mo to infiltrate into the lung tissue.

We thus compared the phenotype of infiltrated and vascular Ly6C^high^ Mo in lungs 48 h after secondary infection in sham- and CLP-operated mice (Figure 5C). Representative overlays (upper panels) and mean fluorescence intensity analysis (lower panels) revealed that in sham- or CLP-operated mice, all activation markers tested were more intensively expressed on infiltrated (CD45vivo^−^) compared to on vascular (CD45vivo^+^) Mo. However, both vascular and infiltrated Mo from septic mice displayed severely reduced expression of MHC-II and of the co-stimulation markers CD80, CD86, and PDL-1. Globally, these data indicate that the increased susceptibility and severity of septic mice to secondary infections is associated to a defective cytokine environment and a limited Mo activation rather than to an altered Mo infiltration.

### In Septic Mice, Infiltrated Ly6C^high^ Mo Display Defective Phagocytic Activity

**Figure 6:**
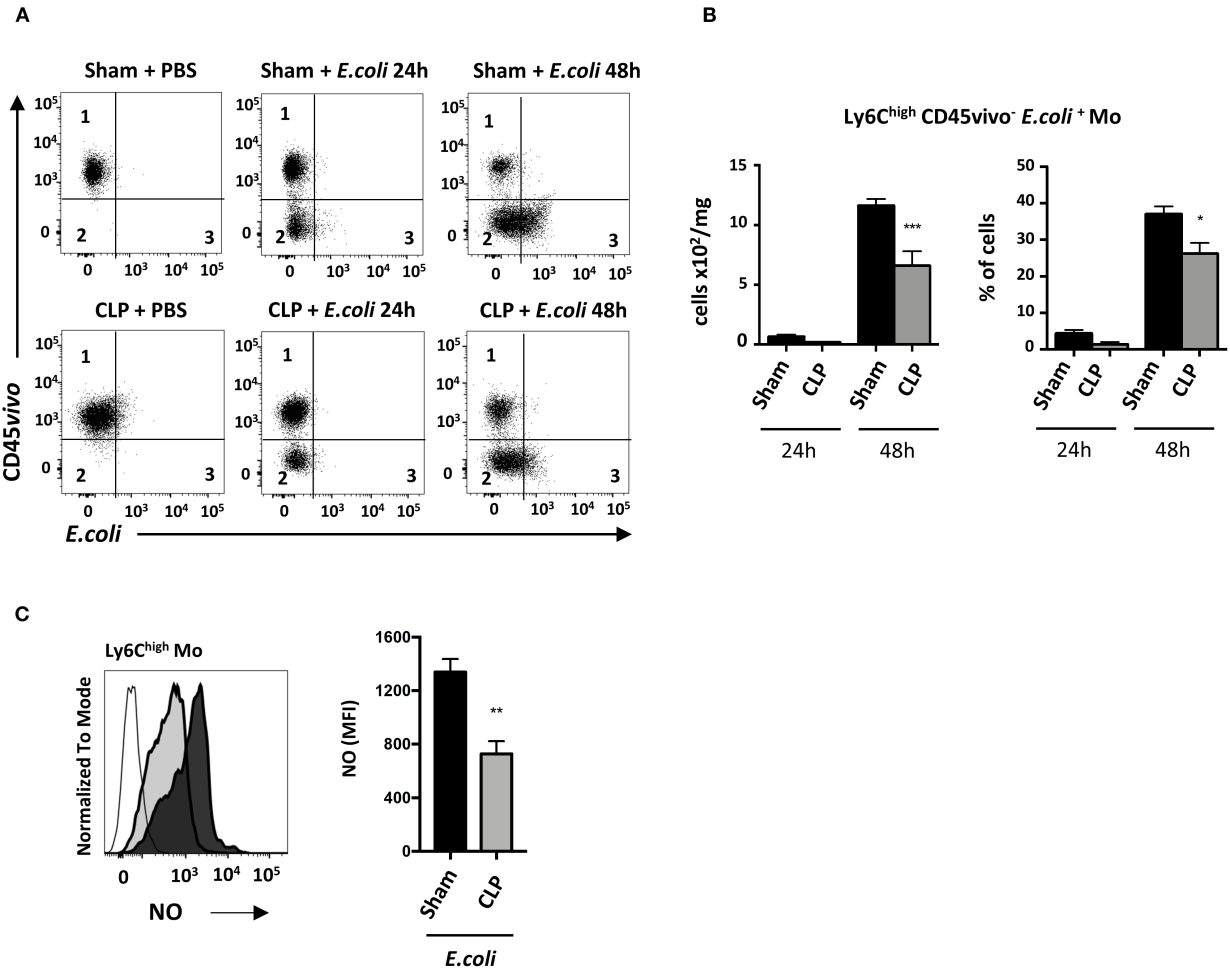
In septic mice, infiltrated Ly6C^high^ Mo displays defective phagocytic activity. **(A)** Dot plot showing Ly6C^high^ Mo in lung from sham- (upper panels) and CLP- (lower panels) operated mice after PBS (left panels) or 24 h (middle panels) and 48 h (right panels) post *E. coli*. Quadrant 1 represents vascular non-phagocytic cells (CD45vivo^+^ *E. coli*^−^), quadrant 2 identifies infiltrated non-phagocytic cells (CD45vivo^−^ *E. coli*^−^) and quadrant 3 represents the infiltrated phagocytic monocytes (CD45vivo^−^ *E. coli*^+^) in lungs. **(B)** Numbers and proportions of lung infiltrated phagocytic Ly6C^high^ Mo (quadrant 3) at 24 or 48 h post *E. coli* pulmonary infection in sham- and CLP-operated mice (*n* = 8 mice from three repeated experiments). Unpaired Student's *t* test was performed to compare Sham and CLP-operated mice in presence of *E. coli*. **(C)** Representative overlay (left panel) and mean fluorescence intensity of FITC (right panel) for NO production of Ly6C^high^ Mo 48 h post *E. coli* pulmonary infection in sham- (black section) and CLP-(gray section) operated mice by flow cytometry (*n* = 6–8 mice from two repeated experiments). FMO is represented in the overlay by a thick gray line. Unpaired Student's *t* test was performed to compare Sham and CLP-operated mice in presence of *E. coli*. ^*^p < 0.05; ^**^p < 0.01; ^***^p < 0.001.

We next investigated the functional defect of septic Mo. Phagocytic Ly6C^high^ Mo were quantified by flow cytometry (Figure 6A). Twenty-four hours after *E. coli* infection, very few infiltrated Ly6C^high^ Mo (CD45vivo^−^) were associated with *E. coli* staining (CD45vivo^−^ *E. coli*^+^) in sham and CLP-operated mice (middle upper and lower panels). At 48 h (right upper and lower panels), many more Ly6C^high^ Mo were infiltrated and positive for *E. coli* in both control and septic mice. Quantitative analysis (Figure 6B) revealed that E. coli phagocytosis by Ly6C^high^ Mo was reduced, in both absolute number and proportion, in septic mice compared to control mice. The NO production was reduced by about 50% in septic compared to control mice (Figure 6C). Altogether, these data indicate that both Mo phagocytic and antibacterial activities are reduced during late sepsis.

## Discussion

Although initially underestimated, the onset of severe immunosuppression in patients with sepsis is now a well-established phenomenon (1, 32). Most patients now survive the initial inflammatory phase thanks to the timely administration of antibiotics and efficient life-support systems but suffer from prolonged recurrent secondary infections (27). Indeed, their survival is impaired by the increased risk of recurrent infections, heart failure, and additional debilitating conditions (33). Reasons for such deteriorations are multifactorial but post-septic immune alterations are suspected to have a major impact on patient health. Understanding the kinetic of inflammatory events leading to immunosuppression and the development of secondary infections is important for the development of therapeutic strategies.

Here we used a sublethal polymicrobial sepsis murine model followed by bacterial Escherichia Coli-induced pneumonia to investigate the role of Ly6C^high^ Mo cells during the recovery phase following acute sepsis. Our study revealed a two-step Mo deployment after CLP; the first wave was associated with early acute clinical events (severe weight loss and death) and the second appeared much later during the clinical recovery of the mice, with no obvious signs of disease. Indeed, CLP triggered an early and transitory mobilization of Mo and PMN in the lungs. During the recovery phase, a secondary deployment of Mo and PMN was observed in all non-lymphoid organs studied. This secondary wave was of larger amplitude and lasted longer than the primary wave of the acute phase. Previous works had already demonstrated a strong mobilization of myeloid cells CD11b^+^GR1^+^ in the blood and the bone marrow 1 week post CLP (34). Interestingly, we demonstrated that the deployed Mo remained exclusively localized to the vasculature of the lungs without accumulating in the lung parenchyma. Anti-inflammatory drugs, but not the antibiotic treatment, abrogated Mo mobilization to the lungs, showing that this accumulation was driven by persistent inflammatory signals independent of the infectious state of the mice. This phenomenon was observed in the blood and in several organs (if not all). These data and the myeloid redistribution indicate that even though the mice recovered from the acute infection and appeared healthy, they were still affected at the cellular level by the primary bacterial exposure. The reason for this recurrent chronic inflammation that leads to a secondary, stronger wave is unclear, but it may be associated with a systemic activation caused by organ failure occurring as a consequence of the first infection.

Studies have shown that Ly6C^high^ Mo are involved in controlling inflammation caused by gram-negative pneumonia and abdominal infections (35, 36). Accordingly, our previous work uncovered that Ly6C^high^ Mo in the kidney play a protective role during the early phase of sepsis, involving anti-inflammatory pathways such as IL1-RA (15). However, the role of Ly6C^high^ Mo in lungs during late sepsis had not been described until now. The role of Mo margination to the vasculature of non-lymphoid organs during the recovery phase remained unclear. However, the genetic deletion of CCR2 blocks Mo egress from the BM and the deletion of CX3CR1 impairs their retention in the lung vasculature, hence both reducing the number of marginating Mo (37, 38) in the lungs. These deficiencies are associated with increased death rates, which would argue in favor of a protective role of Mo also during the later phase. Alternatively, Mo accumulation in the lung capillaries could reflect a bystander effect of the sepsis-induced monocytosis. Live imaging of explanted lungs suggested that ECFP monocytes trapped in the lung capillaries were relatively sessile either in Sham- and CLP-operated mice, indicating no change in their migratory behaviors during sepsis. Our results showed that the adoptive transfer of Ly6C^high^ Mo from septic mice to CCR2-deficient mice reduced organ failure and improved survival less efficiently than Mo from sham-operated mice, suggesting that the protective role is impaired in Mo from CLP-surviving mice. Several function markers on Mo are associated with their defect to efficiently control inflammation but also to contain potential secondary infection here caused by *E. coli* airway inhalation. The immunocompromised phase in sepsis is often associated with endotoxin tolerance (31), arguing that initial bacterial endotoxin activation (here the exposition to the fecal microbiota induced by the CLP surgery) renders cells unresponsive when rechallenged with LPS. Our data showed that Ly6C^high^ Mo from septic mice were fully responsive to LPS in terms of cytokine production and surface markers, suggesting a limited endotoxin tolerance effect. A decrease in the expression of MHC-II and CX3CR1 has been described and asscociated with defective Mo activation and sepsis severity (39, 40). The increased expression of integrin CD11b is also consistent with a margination of cells to the vascular endothelium (41). In our results, the cytokine profiles of the lung environment showed an increase of *Ccl2* and *Il4* transcripts, 10 days after sepsis. Unsurprisingly, CCL2 is involved in Mo mobilization and IL-4 participates in T cell polarization toward a Th2 phenotype (34). After secondary infection, transcript production for iNOS, IL-6, and IL-10 was increased while *Il4* was strongly reduced in sham and CLP-operated mice. Previous studies have shown a weakened lung bacterial clearance in two-hit models of sepsis (42, 43). We observed a smaller increase of *iNos* and *Il6* transcripts for septic mice compared to sham-operated mice after the secondary infection. Again, iNOS and IL-6 are involved in the phagocytic function of cells and in bacterial clearance (44, 45). Thus, these molecules may be linked to the impaired Mo function and the increased susceptibility to infection of the mice.

Wolk et al. showed that decreased expression of MHC-II and CD86 on Mo after LPS stimulation limited their ability to induce T-cell proliferation (46). In addition, immune tolerance during septic shock has been associated with abnormalities of the costimulatory pathway (47). Interestingly, we identified a decrease in PDL1 expression on infiltrated septic Ly6C^high^ Mo compared to sham Mo, whereas we did not observe any difference in PD1 expression on any of the cell types studied (data not shown). Nevertheless, blocking PD1 or PDL1 inhibitory signals are shown to be beneficial for survival in murine sepsis models (48, 49). This can also be observed in septic patients where the expression of PD1 and PDL1 by Mo is increased, but more modestly in those who do not survive (50). Recent work from Bianchini et al. shows that PDL-1 identifies non-classical Mo (here Ly6C^low^ Mo) and regulates T cell survival in tertiary lymphoid organs (51). Here we observed that PDL-1 expression was upregulated on Ly6C^high^ Mo while infiltrating the alveolar space upon *E. coli* infection. It is possible that a higher expression of PDL1 on Mo participate in an infectious context to the fine regulation of the immune response. Whether a beneficial effect of PDL-1 blockade is carried out by infiltrating classical or non-classical Mo remains to be explored. Our results indicate that an alteration of the balance between costimulatory and regulatory pathways could participate in the dysregulation of the innate immune response leading to the inefficient control of secondary infections, and could also reduce its efficacy in protecting against tissue dysfunction.

Overall, we conclude that during the recovery phase following acute sepsis, a recurrent systemic inflammation independent of the infectious status occurs. This inflammatory process leads to a long-term deployment of functionally impaired inflammatory Mo to the vasculature of non-lymphoid organs, which fail to favor lung recovery and protection against secondary infections.

## Data Availability Statement

All datasets generated for this study are included in the article/Supplementary Material.

## Ethics Statement

All experiments and protocols were approved by the Comité d'éthique en expérimentation animale Charles Darwin N°5 under the agreement of the French Ministère de l'Education Nationale, de l'Enseignement Supérieur et de la Recherche with the number APAFIS#4369-2016030218219240 v3.

## Author Contributions

CB, BC, AB, and CC designed the study. CB, PH, ML, NG, PL, AM-K, PD, SB, and AB performed experimental work. CB, AB, and CC performed data analysis, developed figures, and wrote the manuscript. AB, BC, and CC provided financial support. All authors contributed in reviewing the manuscript.

## Conflict of Interest

The authors declare that the research was conducted in the absence of any commercial or financial relationships that could be construed as a potential conflict of interest. The reviewer MR declared a past co-authorship with several of the authors to the handling editor.

## Supplementary Material

Supplementary Figure 1 Effect of polymicrobial sepsis on body weight, survival, and kinetics of alveolar macrophages. **(A)** Percentage of body weight loss in CLP (dotted line) and Sham-operated mice normalized to 100%. Animal weight was measured before surgery and on day 1, 2, 3, 7, and 10. **(B)** Survival of sham- or CLP- operated *Cx3cr1*^gfp/+^ mice after surgery. The survival study was carried out on 25 mice for each group. **(C)** Numbers of Alveolar Macrophage (AM) determined by flow cytometry at different time points after CLP. The time zero was defined based on the cell number obtained in sham-operated mice. Each time point represents at least three independent experiments run with 6 to 12 mice.

Supplementary Figure 2 Neutrophil (PMN) and alveolar macrophage (AM) numbers are not impacted by treatments and CX3CR1 or CCR2 deletion. **(A)** Numbers of PMN in the blood, lungs, and bone marrow determined by flow cytometry in sham and septic mice, 10 days after CLP and treatments. Splenectomies were performed a few minutes before the CLP. Antibiotic or Dexamethasone treatments were injected 24 h after CLP. Values represent the mean +/– sem of 10 mice per group from two independent experiments. **(B)** Numbers of Ly6C^low^ in the lungs determined by flow cytometry in sham and septic mice, 10 days after CLP and antibiotic treatments. **(C)** Numbers of PMN and d-AM in the blood, lung and bone marrow determined by flow cytometry, 10 days after sham- and CLP-operated WT (*n* = 10), *Ccr2*^−/−^ (*n* = 6) and *Cx3cr1*^−/−^ (*n* = 8) mice from at least two independent experiments.

Supplementary Figure 3 Late-expanded myeloid cells remain intravascular in the lung of septic mice. **(A,B)** Proportion of Ly6C^low^ CD45vivo+ Mo and PMN CD45vivo+ in lungs 10 days after sham- or CLP-operated mice.
