# Supplementary material for: Sepsis Triggers a Late Expansion of Functionally Impaired Tissue-Vascular Inflammatory Monocytes During Clinical Recovery
Source: Front Immunol. 2020 Apr 30;11:675. doi: 10.3389/fimmu.2020.00675 (PMC7212400; doi:10.3389/fimmu.2020.00675)
Supplement: Supplementary Figure 1 — Effect of polymicrobial sepsis on body weight, survival, and kinetics of alveolar macrophages. (A) Percentage of body weight loss in CLP (dotted line) and Sham-operated mice normalized to 100%. Animal weight was measured before surgery and on day 1, 2, 3, 7, and 10. (B) Survival of sham- or CLP- operated Cx3cr1gfp/+ mice after surgery. The survival study was carried out on 25 mice for each group. (C) Numbers of Alveolar Macrophage (AM) determined by flow cytometry at different time points after CLP. The time zero was defined based on the cell number obtained in sham-operated mice. Each time point represents at least three independent experiments run with 6 to 12 mice. [file Presentation_1.PPTX]

## Slide 1
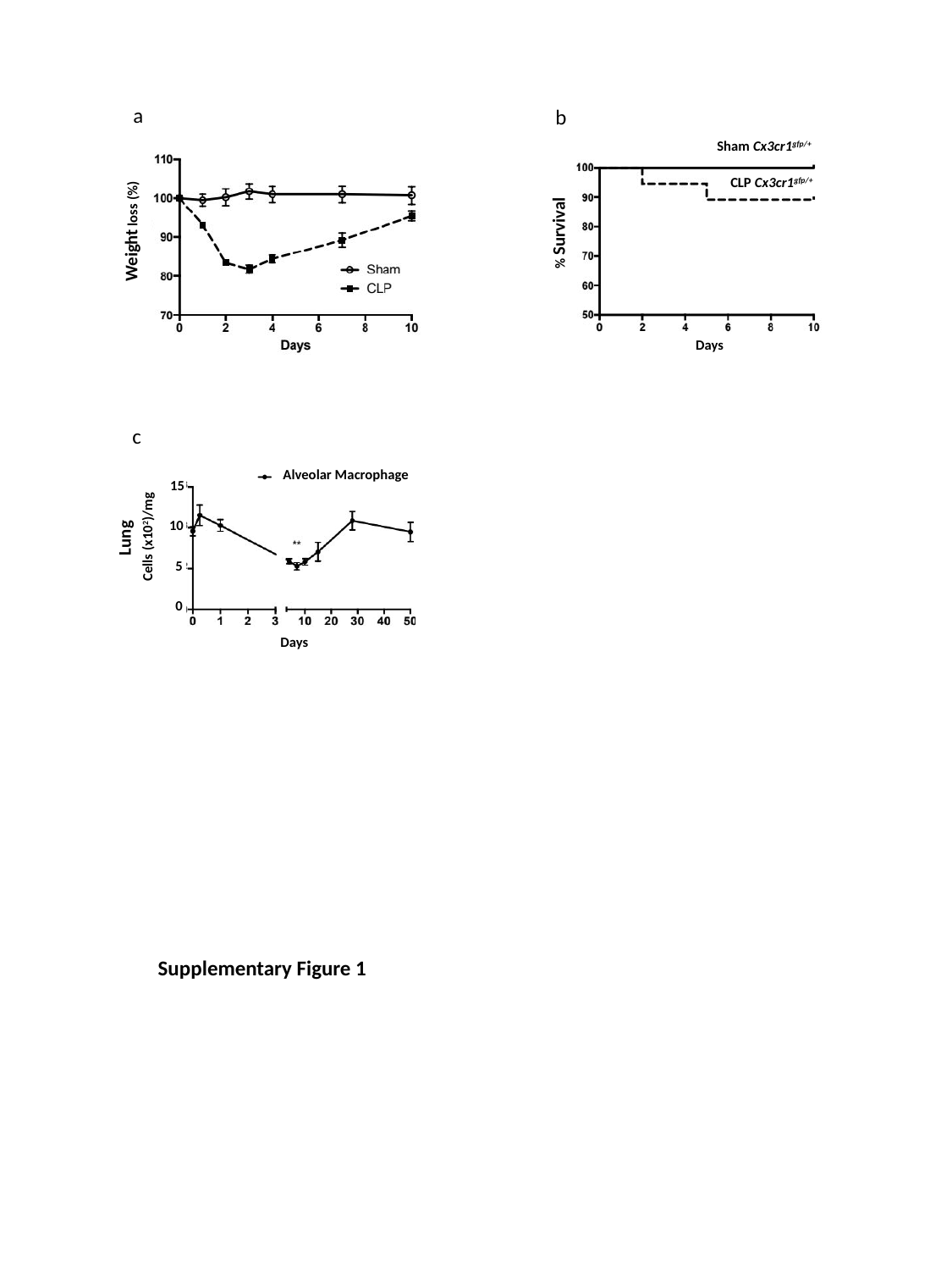

a
b
Sham Cx3cr1gfp/+
CLP Cx3cr1gfp/+
% Survival
Days
Weight loss (%)
c
Alveolar Macrophage
15
10
5
0
Days
Lung
Cells (x102)/mg
Supplementary Figure 1

## Slide 2
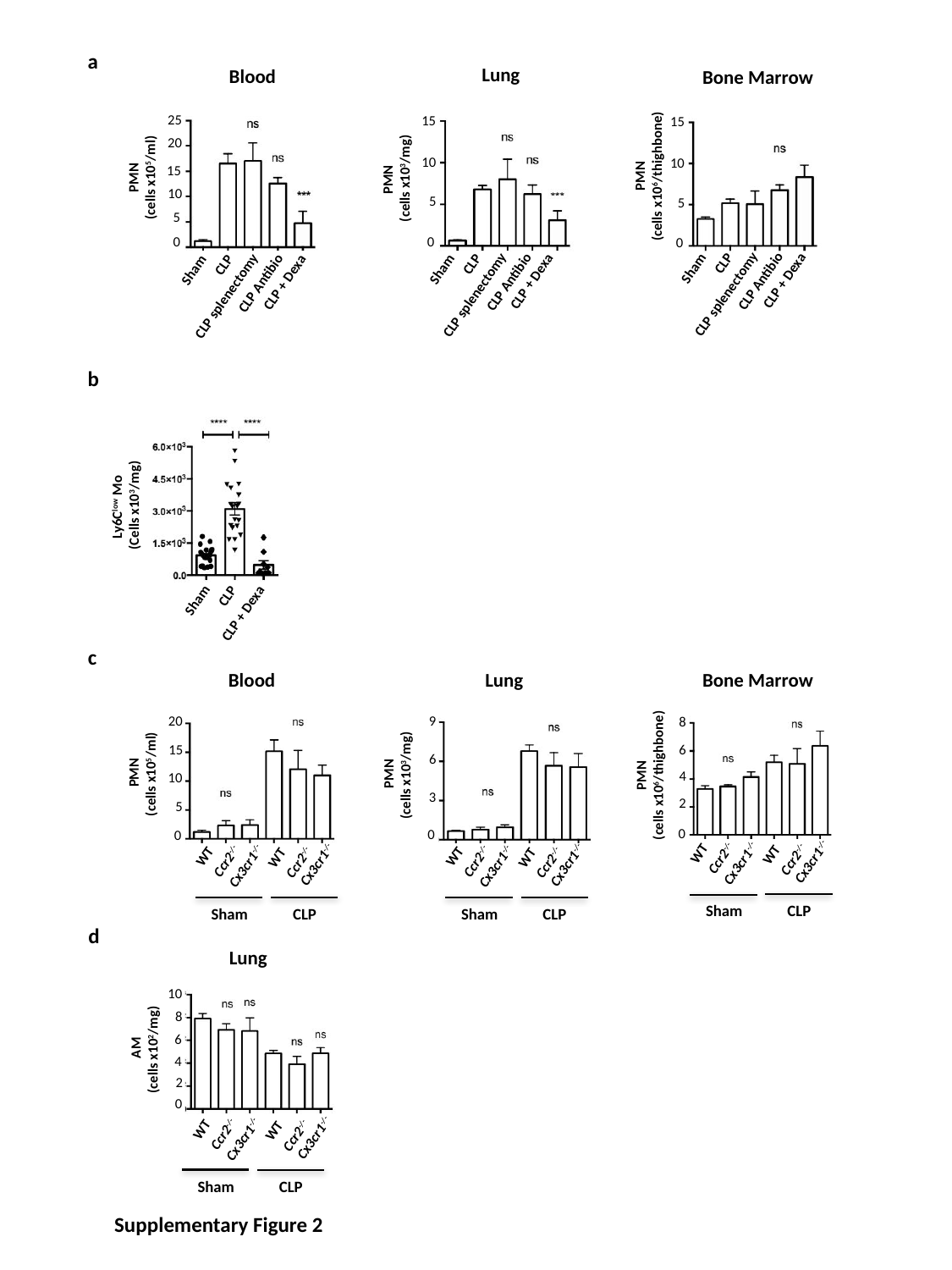

a
Lung
PMN
(cells x103/mg)
CLP
Sham
CLP + Dexa
CLP Antibio
CLP splenectomy
Blood
Bone Marrow
25
15
15
20
10
10
PMN
(cells x106/thighbone)
PMN
(cells x105/ml)
15
10
5
5
5
0
0
0
CLP
Sham
CLP + Dexa
CLP Antibio
CLP splenectomy
CLP
Sham
CLP + Dexa
CLP Antibio
CLP splenectomy
b
CLP
Sham
CLP + Dexa
Ly6Clow Mo
(Cells x103/mg)
c
Blood
Lung
Bone Marrow
20
9
8
6
15
6
 PMN
(cells x105/ml)
 PMN
(cells x103/mg)
PMN
(cells x106/thighbone)
4
10
3
2
5
0
0
0
WT
Ccr2-/-
Cx3cr1-/-
WT
Ccr2-/-
Cx3cr1-/-
WT
Ccr2-/-
Cx3cr1-/-
WT
Ccr2-/-
Cx3cr1-/-
WT
Ccr2-/-
Cx3cr1-/-
WT
Ccr2-/-
Cx3cr1-/-
CLP
Sham
CLP
Sham
CLP
Sham
d
Lung
10
8
6
 AM
(cells x102/mg)
4
2
0
WT
Ccr2-/-
Cx3cr1-/-
WT
Ccr2-/-
Cx3cr1-/-
CLP
Sham
Supplementary Figure 2

## Slide 3
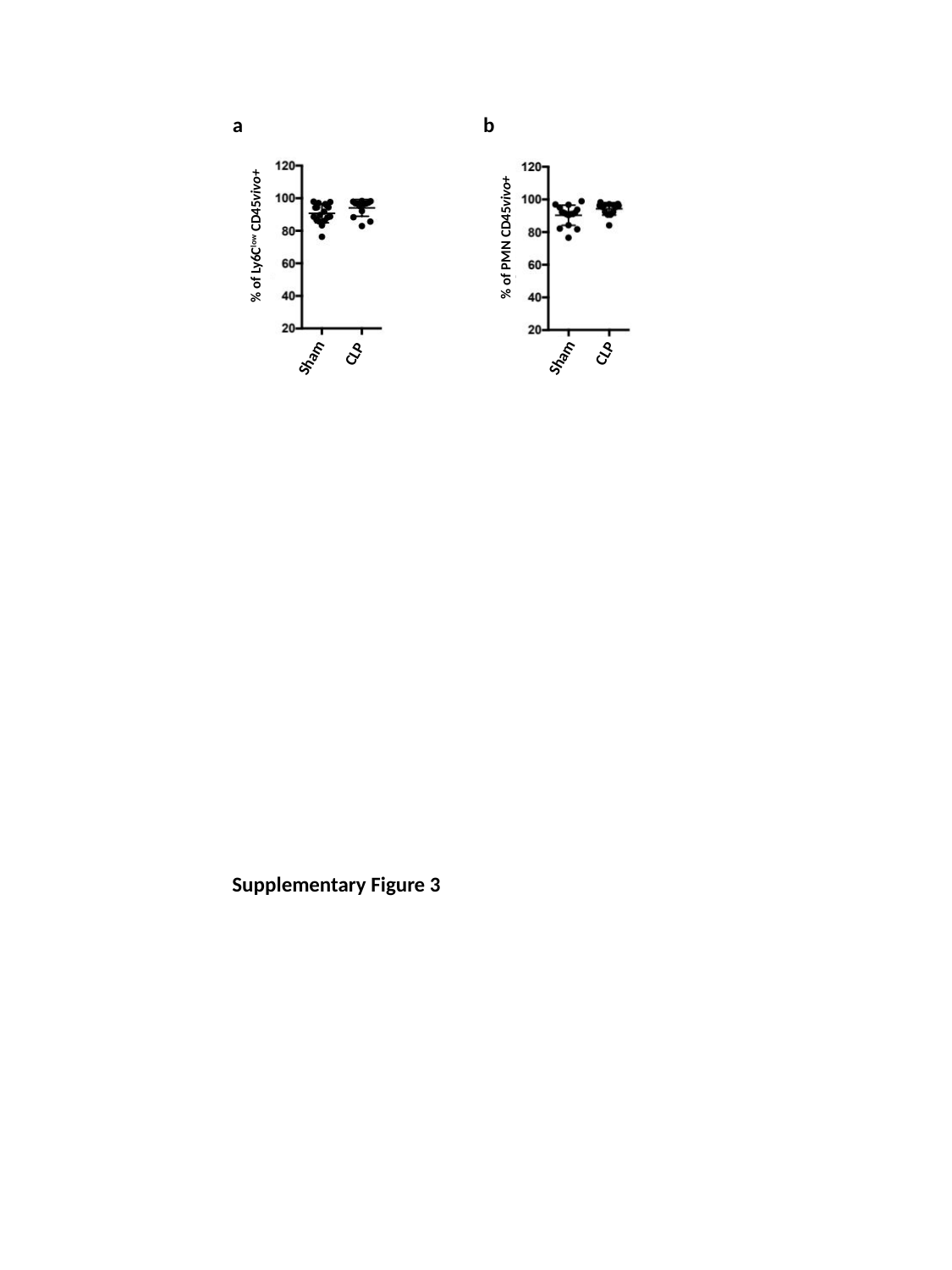

a
b
% of Ly6Clow CD45vivo+
% of PMN CD45vivo+
CLP
Sham
CLP
Sham
Supplementary Figure 3
